# Evolving profile and outcomes of patients with pulmonary arterial hypertension undergoing lung transplantation^1^

**DOI:** 10.1016/j.jhlto.2026.100675

**Published:** 2026-09-03

**Authors:** Pauline Pradère, Marie Charlotte Luong, Jérôme Le Pavec, Justin Issard, Benjamin Coiffard, Sebastien Renard, David Boulate, Benjamin Renaud-Picard, Anne Olland, Thomas Villeneuve, Xavier Demant, Jean Michel Maury, Eva Chatron, Delphine Mitilian, Gaëlle Dauriat, Athénaïs Boucly, Olivier Sitbon, Xavier Jaïs, David Montani, Marc Humbert, Elie Fadel, Olaf Mercier, Delphine Horeau-Langlard, Laurent Savale

**Affiliations:** a Department of Respiratory Medicine, Marie Lannelongue Hospital, Paris Saint Joseph Hospital, Le Plessis Robinson, France; b INSERM UMR-S1358, Pulmonary Hypertension: Pathophysiology and Novel Therapies, Marie Lannelongue Hospital, Le Plessis Robinson, France; c Department of Respiratory Medicine, CHU Laennec, Nantes, France; d Thoracic Surgery Department, Marie Lannelongue Hospital, Paris Saint Joseph Hospital, Le Plessis Robinson, France; e C2VN, INSERM 1263, INRAE 1260, Aix-Marseille University, Marseille, France; f Aix Marseille University, INSERM, INRAE, C2VN, Marseille, France; g Department of Cardiology, Hôpital La Timone, Marseille, France; h Department of Thoracic and Oesophageal Surgery and Lung Transplantation, Aix-Marseille University, APHM, Hôpital Nord, Marseille, France; i Strasbourg Lung Transplant Program, Strasbourg University Hospital, Strasbourg, France; j INSERM UMR 1260, University of Strasbourg, Strasbourg, France; k Thoracic Surgery and Lung Transplantation Department, University Hospital Strasbourg, Strasbourg, France; l Research Unit 1260, Regenerative Nanomedicine INSERM UMR 1260, Université de Strasbourg CRBS, Strasbourg, France; m Department of Respiratory Medicine, University Hospital, Toulouse, France; n INSERM UMR 1291, Toulouse Institute for Infectious and Inflammatory Diseases (Infinity), Université de Toulouse, France; o Department of Respiratory Medicine, Haut-Lévêque Hospital, Bordeaux, France; p Department of Thoracic Surgery, Lung and Heart-Lung Transplantation, Hôpital Louis Pradel, Hospices Civils de Lyon, Lyon, France; q EA 3738 CICLY, Université Claude Bernard Lyon 1, Lyon, France. 14 Physiopathology and Epidemiology of Respiratory Diseases, UMR1152 INSERM and Universite de Paris, Paris, France; r Hospices Civils de Lyon, Hôpital Louis Pradel, Department of Respiratory Medicine, Lyon, France; s Université Paris–Saclay, Inserm UMR_S 1358 (HPPIT), Department of Respiratory and Intensive Care Medicine (FHU André Cournand, ERN-LUNG), Hôpital Bicêtre (AP-HP), Le Kremlin Bicêtre, France

**Keywords:** pulmonary arterial hypertension, doublelung transplantation, heart-lung transplantation, survival

## Abstract

**Background:**

Despite major advances in targeted therapies, lung transplantation remains a key option for selected patients with end-stage pulmonary arterial hypertension (PAH) but is associated with substantial clinical and organizational challenges. Our aim was to describe the impact of changes in advanced PAH management and referral strategies on the profile of transplanted patients and outcomes.

**Methods:**

All patients with PAH (except congenital heart disease), who underwent lung or heart–lung transplantation between 2008 and 2023 were retrospectively studied in France. Data were obtained from the National PAH and Transplant Registries. Patient characteristics, transplant strategies, and outcomes were compared between 2008–2015 (period 1) and 2016–2023 (period 2).

**Results:**

Overall, 241 PAH patients underwent transplantation. The proportion of patients aged ≥50 years increased from 30% to 44% (p=0.04). Time from diagnosis to transplantation tended to increase (39 vs. 46 months, p=0.2). Pre-operative extracorporeal membrane oxygenation (ECMO) as a bridge to transplantation increased from 6% to 14% (p=0.05), while post-operative ECMO use remained stable (35% vs. 36%). Three-month post-transplant survival was 85% (95% CI 78–92%) and 91% (95% CI 86–96%) in periods 1 and 2, respectively. In the PAH population aged ≤ 65 years, the ratio of deaths without transplantation to transplantations performed exceeded 5:1, with marked heterogeneity across aetiologies.

**Conclusion:**

Advances in severe PAH management have enabled transplantation in older and more intensively treated patients without compromising short-term outcomes. However, major disparities in access to transplantation persist across PAH aetiologies, underscoring the persistent challenges in advanced PAH management.

## Introduction

Pulmonary arterial hypertension (PAH) is a severe condition marked by a progressive rise in pulmonary vascular resistance (PVR) leading to right heart failure, and ultimately death. A better understanding of its underlying pathophysiology has led to the development of targeted therapies that have improved exercise capacity, quality of life, and survival.^1^, ^2^ Nevertheless, PAH remains incurable, with mortality rates of 20–30% within three years of diagnosis.^3^, ^4^, ^5^

While medical therapies can stabilize disease progression in many patients, a subset will develop maladaptive right ventricular (RV) remodeling despite maximal treatment.^6^ This progression markedly increases the risk of severe, irreversible RV failure and death in the short to medium term. For these patients, double lung transplantation (DLT), and, in selected cases, heart-lung transplantation (HLT), represents a vital therapeutic option.^7^ However, the unique pathophysiology and specific needs of this population pose significant challenges. Unlike other lung diseases, PAH is associated with distinct prognostic factors that may complicate access to transplantation, with a higher risk of death on the waiting list.^8^ Furthermore, these patients carry a particularly high risk of primary graft dysfunction (PGD) and pulmonary edema, requiring meticulous perioperative management.^7^, ^9^

In recent years, various strategies have been developed to improve the prognosis of PAH patients who are potential candidates for transplantation in most countries.^10^ The use of refined risk stratification tools now allows for earlier identification of patients who should be referred to expert transplant centers and considered for listing.^7^, ^11^, ^12^ In parallel, the development of mechanical circulatory support techniques for patients with RV failure, together with adaptations of organ allocation rules to prioritize those with a high risk of short-term mortality, has contributed to reducing waitlist mortality.^13^ In countries using lung allocation scores (LAS), efforts have also been made to address the historical disadvantage faced by PAH patients in access to transplantation.^14^, ^15^, ^16^, ^17^ Finally, advances in the perioperative management of PAH patients undergoing transplantation have been proposed with the aim of reducing procedure-related mortality and improving early postoperative outcomes.^7^

In France, donor lungs for routinely higher patients are allocated to a specific transplant unit on a rotational basis, where the responsible clinician selects the potential recipient from the waiting list. In 2007, the implementation of a high priority list (HPL) system for patients suffering from acute life-threatening deterioration, aimed to reduce waiting-list mortality.^13^, ^18^

However, since these major developments, including the expansion of medical therapies, new organ allocation rules, and the growing use of mechanical circulatory support for RV failure, new challenges and considerations have emerged. In particular, the evolving profile of PAH patients referred for transplantation in the modern therapeutic era remains insufficiently studied. It is still unclear whether advances in risk stratification strategies have effectively reduced the proportion of patients requiring urgent transplantation. Moreover, the impact of extracorporeal membrane oxygenation (ECMO) on patient outcomes and survival remains poorly understood, whether used as a bridge to transplant or in the immediate post-transplant period.

In this context, the present study aims to provide a comprehensive overview of how the landscape of transplantation for PAH patients has evolved since the introduction of the high priority allocation program. By comparing two distinct periods (2008–2015 vs. 2016–2023), we seek to assess changes in patients’ characteristics at the time of listing, in therapeutic strategies, and in short-term post-transplant outcomes in France.

## Methods

### Patient population

All patients from the French pulmonary arterial hypertension (PAH) registry aged ≥18 years with a diagnosis of PAH who underwent DLT or HLT between January 2008 and December 2023 were included in this analysis, except patients with congenital heart disease (CHD). The exclusion of CHD aimed to ensure a homogenous group of patients in terms of disease pathophysiology, transplant indication, and surgical approach, as CHD-PAH patients often require different transplant strategies (e.g., combined cardiac repair or preferential HLT for complex lesions). The use of the PAH registry ensures precise and reliable data, particularly regarding PAH etiology, as it records only diagnoses established in reference PH centers. Inclusion criteria required a baseline right heart catheterization (RHC) confirming precapillary pulmonary hypertension, defined according to the guidelines applicable during each study period, initially as a mean pulmonary arterial pressure (mPAP) ≥25 mmHg, pulmonary arterial wedge pressure (PAWP) ≤15 mmHg, and PVR >3 Wood units,^19^ and as mPAP >20 mmHg and PVR >2 Wood units since the last 2022 guidelines.^20^

Data from the French PAH Registry were merged with those from the French National Transplant Registry managed by the Agence de la Biomédecine, allowing for a detailed characterization of patients’ care trajectories both before and after transplantation. In parallel, we extracted from the French PAH Registry all patients aged ≤ 65 years who died during the study period with a diagnosis of PAH (excluding CHD) and without a record of LT or HLT, to serve as a comparison group. The age threshold of 65 years was selected as it represents the theoretical upper limit for transplantation eligibility in patients with PAH. Data extraction was completed on February 5, 2025. For analytical purposes, the 16-year study period was arbitrarily divided into two consecutive 8-year intervals to evaluate temporal trends in clinical practice and to ensure enough patients in each period for meaningful statistical analysis.

All data were anonymised and handled in accordance with French data protection regulations (Commission Nationale de l’Informatique et des Libertés, CNIL). Given the retrospective, registry-based design of this study, individual patient consent was not required. The French PAH Registry was established in compliance with French bioethics laws (CNIL no. 842063). The study was conducted in accordance with the principles of the Declaration of Helsinki. The research protocol received ethical approval from the Nantes Ethics Group in the Health Sector (Groupe Nantais d’Éthique dans le Domaine de la Santé, GNEDS) on January 10, 2024 (approval no. 190).

### Graft allocation rules in France

Donor lungs for routinely listed patients are allocated to transplant units on a rotational basis. Within each unit, the responsible clinician selects the potential recipient from the waiting list based on multiple clinical and logistical factors. There are no individual recipient scoring system and no routine national allocation of grafts by patient name. A HPL system was introduced in 2007 to improve access to lung transplantation (LT) for patients with end-stage, life-threatening disease. In these cases, donor lungs are first offered to centers with patients on the urgent transplant list, before being offered to other centers according to the same rotational principle. Requests for priority listing are reviewed by an expert panel, and the vast majority of these requests are approved.

### Statistical analysis

Continuous variables are expressed as median (interquartile range (IQR) 25%–75%). Categorical data are reported as number (n) and proportion (%). Clinical characteristics, comorbidities, risk stratification based on the ESC/ERS four-strata model,^5^ treatments, and transplant outcomes were analyzed across two periods: 2008–2015 and 2016–2023. Statistical comparisons were performed using chi-square and t-tests where appropriate. No multiple imputation was performed. Missing data were handled using complete case analysis, whereby only patients with available data for a given variable were included in the corresponding analysis. The number and proportion of missing values for each variable are reported in the tables. We studied survival on the waiting list and after transplantation with the Kaplan–Meier method and the log-rank test. Univariable and multivariable logistic regression models were performed to assess the association between pre-transplant characteristics of patients, characteristics of the transplant itself and risk of death at 12-months post-transplant. We included in the univariate analysis the variables known or expected to have an impact on this risk of death. The variables with a p-value ≤ 0.05 in the univariable analysis were included in the multivariable model. A p-value < 0.05 was considered statistically significant. All statistical analyses were performed using R studio (Version 2024.04.2+764).

## Results

### Lung transplantation activity and care trajectories in PAH

**Figure 1:**
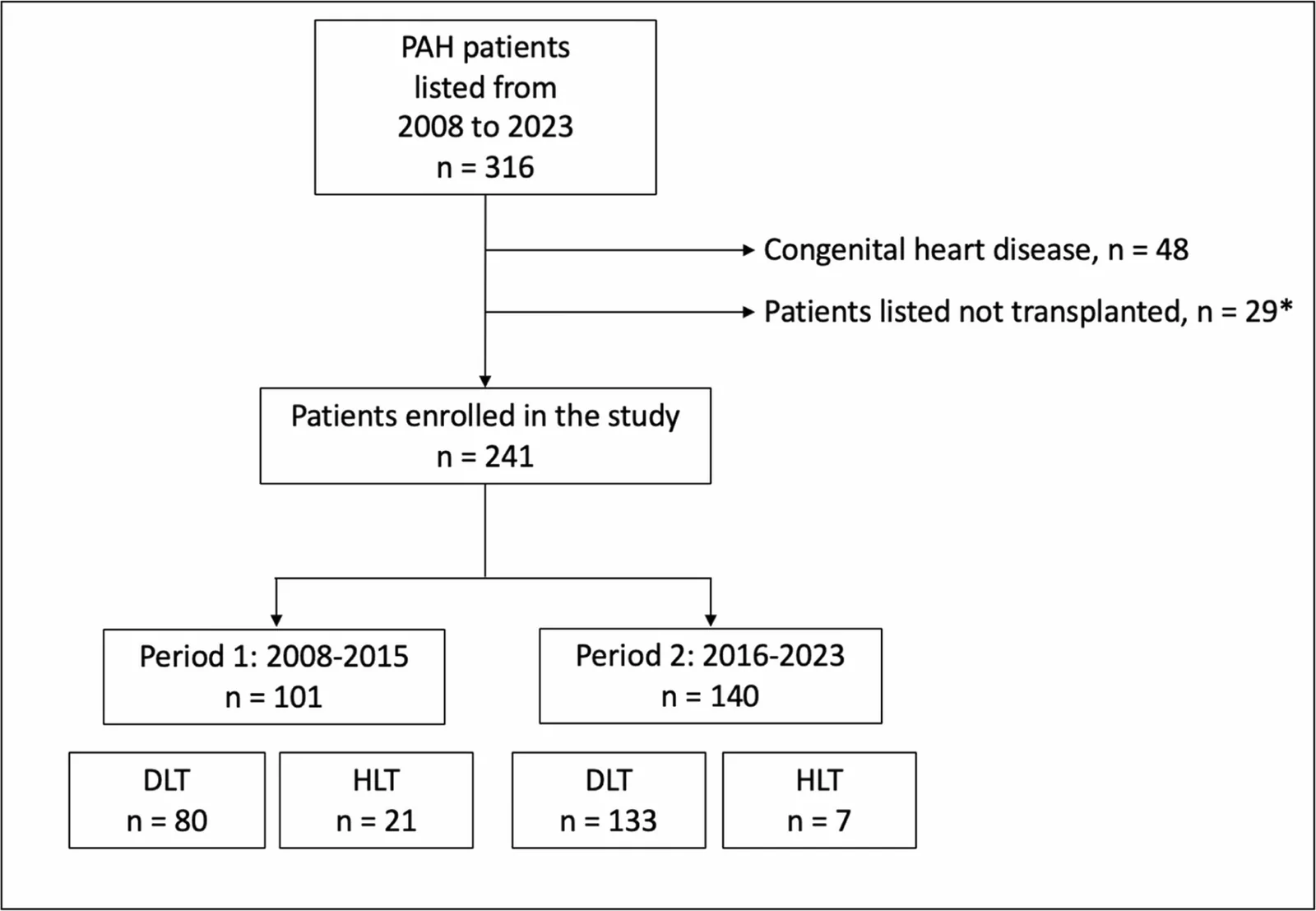
Study flow diagram. *Sixteen patients were listed but not transplanted during period 1: twelve patients died on the waiting list, one patient eventually refused the transplantation, two patients were removed from the list because of PAH improvement and one because of PAH worsening. Thirteen patients were listed but not transplanted during period 2: four patients died on the waiting list, two patients eventually refused the transplantation, one patient were removed from the list because of PAH improvement and one because of PAH worsening, one patient was removed from the list because he developed a major contraindication to transplantation, four patients were still on the waiting list at the time of the study analysis. Abbreviation: PAH for Pulmonary Arterial Hypertension, CHD for Congenital Heart Disease, DLT for Double Lung Transplantation, HLT for Heart-Lung Transplantation.

Between January 2008 and December 2023, a total of 316 patients with PAH and enrolled in the French PAH registry were listed for DLT or HLT. After excluding patients with CHD (n = 48) and patients who were listed but not transplanted (n = 29), 241 patients (213 DLT and 28 HLT) were included in the present analysis. Of these, 101 patients were transplanted during period 1 and 140 during period 2 **(**Figure 1**)**. Three patients were listed during period 1 but were transplanted during period 2. These patients were therefore analyzed in the period 2 cohort.

Only one patient in our cohort was listed but not transplanted because of refusal of the transplantation by the patient several months after listing. This woman, aged 18 years, who suffered from idiopathic PH, was therefore not included in our analysis.

Over the same period, a total of 5012 LT were performed in France, including 2276 during period 1 and 2736 during period 2. Additionally, 191 HLT were performed (123 in period 1 and 68 in period 2). Transplants for PAH accounted for 4.2% and 5.0% of all transplants in periods 1 and 2, respectively (or 5.0% and 5.9% when including patients with CHD).

Over the study period, among the five French centers performing LT for PAH, the majority of procedures were performed at Marie Lannelongue Hospital (Le Plessis Robinson), accounting for 75% of transplants during period 1% and 76% during period 2. The comparison of patient outcomes according to the transplant center is displayed in Table S2.

### Characteristics of PAH patients at time of transplantation

Patient characteristics at the time of transplantation are detailed in Table 1. The proportion of female patients remained consistent across both periods, at 64%. The median age at transplant showed a non-significant increase from 43.8 (IQR, 31.4–52.1) in period 1–47.0 (IQR, 33.8–55.6) years in period 2 (p=0.2), with a notable rise in the proportion of patients aged ≥50 years (30% vs. 44%, p=0.04). The distribution of PAH aetiologies remained stable over time, with idiopathic and heritable forms together representing more than half of the cases. No significant differences were observed between periods regarding the age at diagnosis. However, the median interval between the PAH diagnosis and transplantation tended to lengthen, from 39.2 (IQR, 16.0–72.1) months in period 1–46.5 (IQR, 16.9–99.5) months in period 2 without significant difference (p=0.2). Comorbidities or associated conditions other than PAH remained limited in this selected population.

**Table 1:** Characteristics of Transplanted Patients according to the Period

	**Period 1 2008–2015 n=101**	**Period 2 2016–2023 n=140**	**Missing Data n (%)**	**P Value**
**Age at transplant,** years, median [Q1-Q3]	43.8 [31.4–52.1]	47.0 [33.8–55.6]	0 (0)	0.22
**Age at transplant ≥ 50 years,** n (%)	30 (30)	61 (44)	0 (0)	0.04
**Sex, female,** n (%)	65 (64)	90 (64)	0 (0)	1.00
**BMI, kg/m2,** median [Q1-Q3]	23.0 [21.0–25.0]	24.1 [21.0–27.0]	0 (0)	0.03
**≥ 1 comorbidity**^a^**,** n (%)	35 (35)	67 (48)	0 (0)	0.06
**Past history of smoking,** n (%)	40 (40)	66 (47)	1 (0.7)	0.30
**Type of PAH,** n (%)			0 (0)	0.70
– Idiopathic – Heritable – PVOD – Drug-induced PAH – Connective tissue disease – PoPH – associated with HIV	29 (28) 26 (26) 23 (23) 5 (5) 17 (17) 0 (0) 1 (1)	42 (30) 31 (23) 35 (25) 6 (4) 21 (15) 3 (2) 0 (0)		
**Delay between PAH diagnosis and transplantation, months,** median [Q1-Q3]	39.2 [16.0–72.1]	46.5 [16.9–99.5]	10 (7)	0.21
**Intravenous prostacyclin at the time of listing, n (%)**	67 (68)	83 (60)	4 (2)	0.27
**Triple therapy at the time of listing, n (%)**	42 (42)	78 (56)	0 (0)	0.04

Abbreviations: BMI, body mass index; HIV, human immunodeficiency virus; PAH, pulmonary arterial hypertension; PVOD, pulmonary veno-occlusive disease; PoPH, porto-pulmonary hypertension.

a At least one comorbidity among hypertension, chronic cardiac arrhythmia, coronary artery disease, peripheral vascular disease, splenectomy, psychiatric disorders, diabetes, chronic renal failure, history of venous thrombosis and/or pulmonary embolism, chronic infection (C and/or B hepatitis and/or HIV or Human Immunodeficiency Virus), chronic liver disease, obesity (BMI ≥ 30 kg/m2), history of cancer, hematological disease (benign), associated pulmonary disease other than PAH or than pulmonary embolism

### Disease severity at time of listing

PAH severity at time of listing is detailed in Table 2. The proportion of patients receiving triple combination therapy increased from 42% in period 1–56% in period 2 (p=0.05). No significant changes were observed in haemodynamic parameters assessed by RHC at time of listing during the two periods. Similarly, there were no notable differences in 6-minute walking distance (6MWD) or renal function.

**Table 2:** PAH-related Characteristics at Time of Listing

	**Period 1 2008–2015 n=101**	**Period 2 2016–2023 n=140**	**Missing Data n (%)**	**P Value**
**6MWD, meters,** median [Q1-Q3]	375 [285−468]	370 [300−452]	18 (8)	0.71
**NYHA functional class,** n (%)			5 (2)	< 0.01
– II – III – IV	8 (8) 31 (31) 61 (61)	18 (13) 69 (51) 49 (36)		
Haemodynamic*				
– mPAP, mm Hg, median [Q1-Q3] – PAWP, mm Hg, median [Q1-Q3] – RAP, mm Hg, median [Q1-Q3] – CI, l/min/m2, median [Q1-Q3] – PVR, WU, median [Q1-Q3]	54.0 [45.5–62.0] 8.0 [5.0–11.0] 7.0 [5.0–11.0] 2.7 [2.2–3.3] 10.0 [7.1–12.7]	52.0 [42.3–65.0] 8.0 [6.0–12.0] 7.0 [5.0–11.0] 2.7 [2.2–3.3] 8.8 [6.1–13.4]	4 (2) 9 (4) 9 (4) 8 (3) 9 (4)	0.59 0.17 0.63 0.93 0.24
**Biological variables**				
– Creatinine, micromol/L, median [Q1-Q3] – eGFR, ml/min/m2, median [Q1-Q3] – NTproBNP, ng/L, median [Q1-Q3]	82.5 [69.9–101.3] 75.0 [55.7–92.6] 382 [151–1711]	78.7 [63.0–93.3] 78.7 [60.6–101.1] 936 [270–2242]	41 (17) 41 (17) 102 (42)	0.04 0.14 0.33
**4-strata risk stratification,** n (%)			19 (8)	< 0.01
– Low risk – Intermediate low – Intermediate high – High	4 (4) 60 (64) 26 (28) 4 (4)	17 (13) 59 (46) 51 (40) 1 (1)		

Abbreviations: 6MWD; 6-minutes walking distance, NYHA, New York Heart Association; mPAP, mean pulmonary arterial pressure; PAWP, pulmonary arterial wedge pressure; RAP, right atrial pressure; CI, cardiac index; PVR, pulmonary vascular resistance; WU, Wood Units; eGFR, estimated glomerular filtration rate; PAH, pulmonary arterial hypertension; NT-proBNP, N-terminal pro b-type natriuretic peptide.

* Median interval between right heart catheterization and transplant was 234 days [IQR:96–393] and 207 days [IQR:123–360] during period 1 and 2 respectively.

Risk stratification using the ESC/ERS four-strata model showed that more than 80% of patients were classified as having an intermediate mortality risk at the time of listing. Specifically, intermediate-low risk was observed in 64% of patients in period 1% and 46% in period 2, while intermediate-high risk increased from 28% to 40%. High-risk patients remained rare (4% in period 1% and 1% in period 2). Notably, a higher proportion of patients were listed at low mortality risk in period 2 compared to period 1 (13% vs. 4%, p = 0.005). Among the 21 patients categorized as low-risk at the time of listing, 10 (48%) were diagnosed with pulmonary veno-occlusive disease (PVOD).

### Evolution of transplant procedures

Evolution of transplant procedures are detailed in Table 3. The median waiting time on the conventional transplant list significantly decreased from 194 days (IQR, 90–305) in period 1–103 days (IQR, 36–191) in period 2 (p=0.005). The use of DLT increased markedly, while HLT became rare, with DLT performed in 79% of patients in period 1 compared to 95% in period 2 (p<0.001) (Supplemental Figure 2).

**Table 3:** Comparison of Transplant Procedures between Periods

	**Period 1 2008–2015 n=101**	**Period 2 2016–2023 n=140**	**Missing Data n (%)**	**P Value**
**High priority list,** n (%)	56 (55)	58 (42)	0 (0)	0.05
**Waiting times,** days, median [Q1-Q3]			0 (0)	
− Conventional transplant − High priority list − Overall	194 [90−305] 3 [2–6] 75 [13−221]	103 [36−191] 3 [1–5] 80 [19−165]		<0.01 0.43 0.84
**Evaluated and supported to lung transplant in ICU**1, n (%)	16 (16)	20 (14)	0 (0)	0.92
**ECMO as bridge to transplant**2, n (%)	6 (6)	20 (14)	8 (6)	0.05
**Type of transplant,** n (%)			0 (0)	< 0.01
− Heart-lung transplantation − Double-lung transplantation	21 (21) 80 (79)	7 (5) 133 (95)		
**Post-transplant ECMO**^3^, n (%)	28 (35)	37 (36)	58 (24)	0.99

Abbreviations: 6MWD,6-minutes walking distance; BMI, body mass index; ECMO, extracorporeal membrane oxygenation; HIV, human immunodeficiency virus; ICU, intensive care unit; NYHA, New York Heart Association; PAH, pulmonary arterial hypertension; PH, pulmonary hypertension; PVOD, pulmonary veno-occlusive disease; PoPH, porto-pulmonary hypertension;

1 PH causes in patients evaluated and bridged to lung transplant in the ICU, with no prior pre-transplant assessment were: heritable PAH (n=12, 33%), PVOD (n=10, 28%), idiopathic PAH (n=7, 19%), auto-immune disorders (n=4, 11%), toxic PAH (n=1, 3%), HIV PAH (n=1, 3%), PoPH (n=1, 3%).

2 ECMO as a bridge to transplant corresponds to veno-arterial peripheral ECMO.

3 Post-transplant ECMO refers to intraoperative central or peripheral veno-arterial ECMO and/or to veno-venous ECMO for primary graft dysfunction (with detailed proportions not available in this dataset).

The proportion of patients listed through the HPL system decreased from 55% in period 1–42% in period 2 (p=0.05). In both periods, 16% and 15% of patients, respectively, were evaluated and directly bridged to urgent transplantation in the intensive care unit (ICU) without prior pre-transplant assessment. Waiting times for patients on the urgent list remained unchanged across the two periods. Finally, the use of circulatory support as a bridge to transplantation increased from 6% to 14% between periods 1 and 2 (p=0.05). The proportion of patients managed with post-operative ECMO was similar between the two periods (35% and 36% respectively) (Supplemental Figure 1).

### Survival

**Figure 2:**
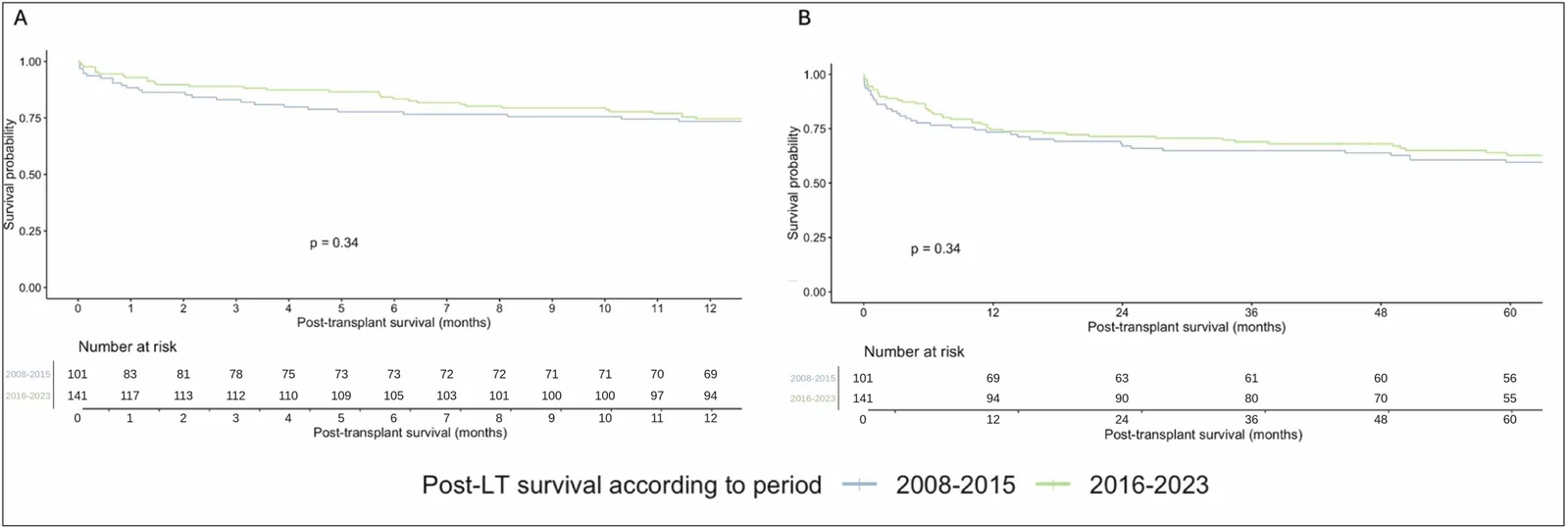
Comparison of 1 and 5-years post-transplant survival during period 1 (2008–2015) and period 2 (2016–2023). Abbreviation: LT for lung transplantation.

No patients were lost to follow-up at one year after transplantation. Post-transplant survival at 3 months was comparable between period 1 and period 2, with survival rates of 85% (95% CI: 78–92%) and 91% (95% CI: 86–96%), respectively (p=0.1). 12-month survival rates were 75% (95% CI: 67–84%) and 76% (95% CI: 69–83%), respectively (p = 0.1) (Figure 2). In univariate analysis, higher age, BMI, and COMPERA 2.0 score at time of listing were associated with an increased risk of death at one-year post-transplant **(**Table 4**)**. Age at listing was the only factor that remains significant in multivariable analysis **(**Table 4**).** A comparison of one and five years post-transplant survival between PAH patients and those transplanted for other lung diseases is presented in the Supplemental Table 1. PAH is associated with the highest 12-month mortality risk in period 2. Twelve-month survival was 76% [69–83%], compared with 80% [77–82%], 86% [84–88%], and 88% [84–90%] for patients with lung fibrosis, COPD-emphysema, and cystic fibrosis respectively (p<0.001).

**Table 4:** Univariable Logistic Regression for Death at 12-months Post-transplant

	**Univariable**		**Multivariable**	
	**OR (95% CI)**	*p Value*	**OR (95% CI)**	*p Value*
**Male**	0.72 (0.38–1.33)	*0.33*		
Age*	1.04 (1.02–1.07)	*0.002*	1.04 (1.01–1.07)	*0.02*
**Age ≥ 50 years**	2.05 (1.14–3.70)	*0.02*		
BMI, kg/m2*	1.06 (1.00–1.13)	*0.05*	1.01 (0.93–1.09)	*0.83*
**Past history of smoking**	0.66 (0.36–1.18)	*0.23*		
**Connective tissue disease**	1.90 (0.89–3.96)	*0.09*		
eGFR	0.99 (0.98–1.01)	*0.34*		
eGFR < 60 ml/min/1.73m^2^	1.81 (0.84–3.79)	*0.12*		
4-strata risk score*	1.73 (1.10–2.77)	*0.02*	1.50 (0.89–2.58)	*0.13*
**High priority list**	1.47 (0.83–2.64)	*0.24*		
**Pre-transplant ECMO**	1.00 (0.34–2.55)	*>0.9*		
**Post-transplant ECMO**	1.23 (0.62–2.46)	*0.51*		

Abbreviations: BMI, body mass index; ECMO, extracorporeal mechanical oxygenation, eGFR estimated glomerular filtration rate.

* Variables that were included in the multivariable logistic regression analysis.

### Comparison of transplanted patients with patients that died during the study period

**Figure 3:**
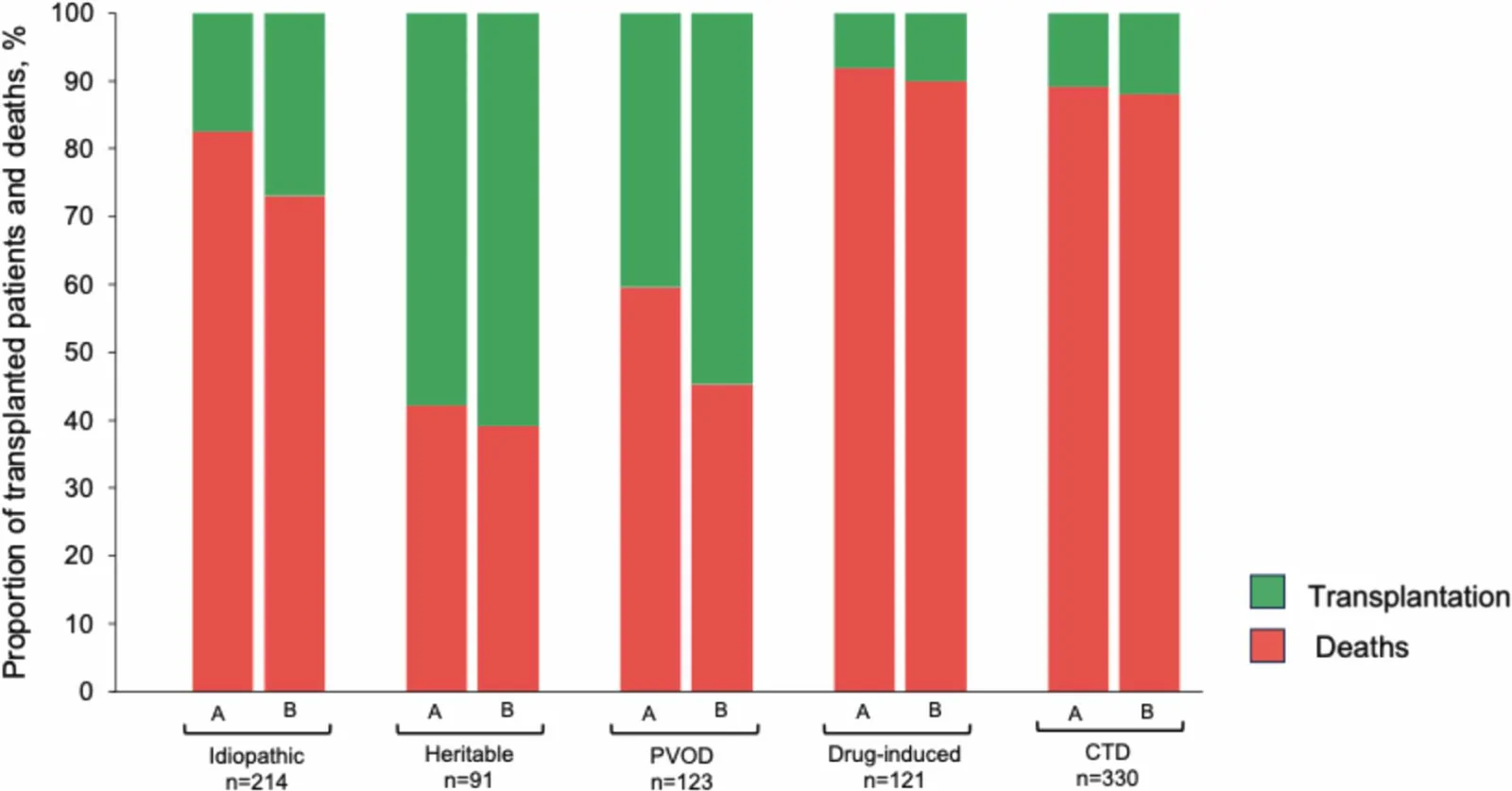
Ratio of transplanted patients to deceased patients by period and type of PAH. Abbreviation: CTD, connective tissue disease; PVOD, pulmonary veno-occlusive disease. A corresponds to period 1 (2008–2015) and B to period 2 (2016–2023).

We compared PAH patients who underwent LT/HLT between 2008 and 2023 with all patients recorded in the National PAH Registry (excluding those with CHD) who died at or before the age of 65 during the same period. Overall, 1322 deaths and 241 lung transplants were identified, corresponding to a ratio of more than five deaths for every transplant performed. This ratio varied markedly across PAH subtypes, ranging from one transplant for nine deaths in connective tissue disease (CTD)–associated PAH to 1.5 transplants for one death in heritable PAH **(**Figure 3**)**. As expected, patients with portopulmonary hypertension (PoPH) did not undergo LT/HLT, except for three cases in whom congenital portosystemic shunts had been corrected.

Patients who died were more frequently male, had a higher body mass index (BMI), were more often smokers, presented with a greater burden of comorbidities, and were older at the time of PAH diagnosis **(**Table 5**)**. The cause of death was available for 636 patients (48%). PAH was the leading cause of death in 302 cases (47%), followed by cancer (98 cases, 15%), sepsis (85 cases, 13%), liver disease (69 cases, 11%), lung diseases other than PH (42 cases, 7%), and cerebrovascular disease (18 cases, 3%).

**Table 5:** Comparison of Transplanted Patients Versus PAH Patients Aged ≤ 65 Years Who Died Between 2008 and 2023

	**Deceased Patients 2008–2023 (n=1322)**	**Transplanted Patients 2008–2023 (n=241)**	**Missing Data n (%)**	**p Value**
**Age at PAH diagnosis,** years, median [Q1-Q3]	51 [44−57]	44 [32–54]	0 (0)	*< 0.001*
**Age at outcome**^1^**,** years, median [Q1-Q3]	56 [49–60]	45 [32–54]	0 (0)	*< 0.001*
**Sex, female**, n (%)	662 (50)	155 (64)	0 (0)	*< 0.001*
**BMI**, kg/m2, median [Q1-Q3]	25.6 [21.8–30.5]	23.8 [21.0–26.8]	5 (0.3)	*< 0.001*
**Type of PAH**, n (%)			0 (0)	*< 0.001*
− Idiopathic − Heritable − PVOD − Drug-induced − Connective tissue disease − PoPH − HIV	243 (18) 34 (3) 65 (5) 110 (8) 292 (22) 448 (34) 130 (10)	73 (30) 57 (24) 58 (24) 11 (5) 38 (16) 3 (1) 1 (<1)		
**Addiction**				
− Past history of smoking, n (%) − Past history of addiction, n (%)	803 (61) 73 (6)	107 (44) 1 (<1)	1 (<0.1) 0 (0)	*< 0.001* *0.001*
**Comorbidities**			0 (0)	
− ≥ 1 comorbidity^2^, n (%) − Number of comorbidities, median [Q1-Q3] − Obesity, n (%) − Hypertension, n (%) − Coronaropathy, n (%) − Peripheral vascular disease, n (%) − Chronic kidney disease, n (%) − Diabetes, n (%) − Chronic hepatic disease, n (%) − Psychiatric condition, n (%) − Past history of cancer, n (%)	940 (71) 1 [0−2] 348 (27) 355 (27) 77 (6) 65 (5) 64 (5) 181 (14) 154 (11) 107 (8) 132 (10)	102 (42) 0 [0−1] 30 (12) 38 (15) 3 (1) 3 (1) 4 (2) 17 (7) 2 (1) 9 (4) 11 (5)		*< 0.001* *< 0.001* *< 0.001* *< 0.001* *0.004* *0.02* *0.02* *0.002* *< 0.001* *0.02* *0.01*

Abbreviations: BMI, body mass index; PAH, pulmonary arterial hypertension; PVOD, pulmonary veno-occlusive disease; PoPH, porto-pulmonary hypertension; HIV, human immunodeficiency virus.

1 Transplantation or death

2 At least one comorbidity among hypertension, chronic cardiac arrhythmia, coronary artery disease, peripheral vascular disease, splenectomy, psychiatric disorders, diabetes, chronic renal failure, history of venous thrombosis and/or pulmonary embolism, chronic liver disease, obesity (BMI ≥ 30 kg/m2), history of cancer, hematological disease (benign), associated pulmonary disease other than PAH or than pulmonary embolism

## Discussion

This study provides a national overview of LT for PAH in France by combining data from the French PAH Registry and the French National Transplantation Registry. This unique dataset allows a detailed description of transplanted patients and their care trajectories. Over the 16-year study period, major changes occurred in PAH management, including innovations in therapeutic strategies, and substantial evolution in transplant practices. Among remarkable results to underline, the absolute numbers of DLT increased by 40% while the proportion of HLT decreased by 4 folds. The use of extracorporeal life support as a bridge to transplantation progressively increased, and transplant allocation strategies were refined through the maturation of the national HPL program. Despite a progressive shift toward older transplant candidates with more complex profiles, early post-transplant outcomes remained stable over time. In particular, no significant improvement or deterioration in mortality during the first 12 months after transplantation was observed.

During the study period, the annual number of DLT and HLT in France increased markedly, rising from 215 procedures in 2008–393 in 2019, just before the COVID-19 pandemic (+83%). Despite this substantial expansion in overall transplant activity, PAH consistently represented approximately 5% of transplant indications, suggesting that access to transplantation for this population remained proportionally stable over time. However, the ratio of deaths to transplants among patients with PAH younger than 65 years remained strikingly high, exceeding 5:1, with pronounced heterogeneity across PAH subtypes. Patients who died were more often male, older at PAH diagnosis, and more likely to have a higher BMI, multiple comorbidities, and a history of smoking. These characteristics are known to either limit transplant eligibility or delay referral for transplant evaluation. Interestingly, patients with idiopathic PAH had worse outcomes compared to those with heritable PAH and PVOD. This result may reflect the complex interplay between disease severity, age at diagnosis, and referral timing across PAH subtypes. Patients with PVOD are known to have a particularly poor prognosis without transplantation and are therefore referred more promptly for transplant evaluation, which may paradoxically result in better post-listing outcomes compared to idiopathic PAH patients, who may be managed medically for longer before referral. Similarly, the younger age at diagnosis and the systematic availability of genetic counseling in France for heritable PAH may facilitate earlier identification and referral of these patients, potentially explaining their more favorable outcomes compared to idiopathic PAH.

Transplantation in patients with CTD was very rare, likely reflecting the frequent exclusion of these patients from transplant eligibility due to the systemic nature of CTD, which produces extra-pulmonary manifestations associated with increased post-transplant morbidity and mortality. However, with careful patient selection, post-transplant survival in these patients can be comparable to that of patients without CTD.^21^ Our findings are consistent with a recent study by Kolaitis et al. examining referral patterns for LT in PAH within the United States healthcare system using an allocation score–based approach. In that study, fewer than 12% of patients in the overall PAH population were referred for transplantation, a proportion that increased to only 18–29% among patients in WHO functional class IV or at high risk of mortality. Notably, only 30% of referred patients ultimately underwent transplantation, and comorbidities were again a major determinant of lower referral rates.^22^

Our findings suggest that therapeutic advances have contributed to modestly delaying the need for transplantation. Over time, the proportion of recipients aged 50 years or older has increased, which may reflect greater confidence among clinicians in transplanting older patients, supported by improvements in perioperative management and postoperative care. Nevertheless, age remained a significant predictor of one-year post-transplant mortality in our cohort, underscoring its continued importance despite therapeutic progress. The broader use of triple combination therapy, which became available around 2015–2016, likely played a role in extending the period during which patients can be medically managed before transplantation. Given that median survival in PAH is now estimated at over 7 years, our study period may prevent us from capturing the full impact of these therapeutic advances. More recently, new therapies targeting alternative signaling pathways, such as those involving the TGF-β superfamily, are emerging. Sotatercept, in particular, has generated considerable interest by reducing pulmonary vascular resistance, improving exercise capacity and reducing morbidity and mortality in high-risk patients.^23^ These innovations may further modify the clinical profile of patients with PAH and could prolong survival without the need for transplantation in the coming years. In our cohort, one patient was recently removed from the waiting list due to clinical improvement under sotatercept.

Among patients with PAH, the need for HLT has become increasingly rare. The remaining indications correspond to massive pulmonary artery dilation and/or associated or suspected left heart cardiomyopathy. This trend reflects the recognized ability of the right ventricle to recover after DLT. Importantly, this recovery is now often supported by ECMO in the immediate post-transplant period. In our cohort, approximately one-third of patients required postoperative ECMO to facilitate RV adaptation to the sudden drop in PVR. Some centers have even adopted a more systematic strategy, maintaining ECMO prophylactically after DLT for PAH to promote controlled hemodynamic transition and reduce the risk of early graft dysfunction.^24^ As a result, HLT is now performed predominantly in patients with complex CHD, for whom isolated LT is insufficient. These major haemodynamic challenges associated with the predominant use of DLT rather than HLT did not result in increased post-transplant mortality. Nevertheless, transplantation for PAH remains associated with higher short-term mortality compared with transplantation for other lung diseases, as previously described.^25^, ^26^ The better outcomes observed in lung fibrosis and COPD-emphysema likely reflect recent advances specific to these indications, including the use of veno-venous ECMO as a bridge to transplantation in acute exacerbations of IPF, the reduction in corticosteroid use in IPF management, and the growing preference for double lung transplantation in both fibrosis and COPD. These advances have no equivalent in PAH transplantation, where perioperative management remains particularly challenging.^27^, ^28^

During the study period, the proportion of patients requiring an emergency transplant decreased significantly, possibly reflecting improvements in risk stratification and a more accurate determination of the optimal timing for referral and listing. Previous international guidelines recommended listing patients only once they had reached a high predicted mortality, a threshold that often proved too late and concerned only a minority of candidates.^29^ More recently, expert opinion from the world symposium on PH has shifted toward listing patients at intermediate–high risk despite maximal medical therapy, a group that indeed increased markedly over time in our cohort.^7^ Nonetheless, a substantial proportion of patients were still listed at intermediate–low or even low risk. This discrepancy between guideline recommendations and real-life practice may be explained by several medical considerations such as PAH etiology (PVOD), specific hemodynamic patterns, the clinician’s longitudinal knowledge of the patient’s disease trajectory, or the occurrence of life-threatening events including haemoptysis or coronary compression. This discrepancy is also explained by therapeutic burden, a key element when referring a patient for pre-transplant evaluation. Indeed, the risk scores used for patient stratification do not account for therapeutic burden. Being classified as low mortality risk under maximal therapy, including intravenous prostacyclin and triple combination therapy, is fundamentally different from achieving the same risk category with minimal treatment, as the former reflects a fragile haemodynamic equilibrium that is highly dependent on ongoing intensive therapy and remains a strong indication for transplant referral, as recommended in the international guidelines. Another key element is access to LT, which depends on each country's allograft allocation system as well as individual patient factors. Patients with PAH may have reduced access to lung allografts due to the high proportion of women in this population, who are generally smaller than their male counterparts, and due to higher rates of HLA sensitization. Despite a reduction in HPL, around 40% of patients in both periods ultimately required transplantation under emergency conditions. Notably, in contrast with other indications for LT, emergency transplantation in PAH does not result in excessive post-transplant mortality, underscoring the exceptional ability of these patients to recover when appropriately supported. The increasingly use of ECMO as a bridge to transplantation has profoundly reshaped the management of critically ill PAH patients.^30^ This strategy stabilizes RV failure and has expanded the therapeutic window, illustrating how essential it is that these patients be managed in expert centers capable of offering advanced support options. In France, this reality has led to a deliberate centralization of care, with approximately 80% of PAH transplants performed in a single surgical center directly associated with the national reference center, acknowledging the high level of expertise required for successful outcomes in this population.^7^ The increased use of ECMO over time did not appear to adversely impact post-transplant survival, a finding consistent with recent reports showing that, although prolonged pre-transplant ECMO is associated with higher waitlist mortality, it is not associated with worse post-transplant survival among patients who reach transplantation.^31^, ^32^Notably, neither pre-transplant ECMO support nor high-priority listing was associated with worse post-transplant survival. These two variables are inherently linked, as ECMO support is for patients experiencing acute life-threatening deterioration of their condition, which constitutes the main criterion triggering high-priority listing in our allocation system. Despite this overlap, this result suggests that a well-coordinated, multidisciplinary strategy combining high-priority listing and ECMO bridging can safely support even the most critically ill PAH patients through transplantation without compromising outcomes.

Our study has several limitations. First, the use of registry-based data inevitably resulted in some missing variables that might have provided additional insights, particularly regarding detailed perioperative management and longitudinal trajectories prior to listing. Second, our study period encompasses the COVID-19 pandemic (2020–2023), which may have affected organ procurement, transplant volumes, and post-transplant outcomes. We observed a tendency toward an increased delay between PAH diagnosis and transplantation during period 2, which is probably attributable to earlier PAH diagnosis rather than to delayed transplantation due to the pandemic, given that there was no major difference in the severity of patients at the time of transplantation between period 1 and period 2. Eventually, our analysis reflects practices specific to the French healthcare system. France has a highly centralized organization for PAH and transplant care, with expert centers, coordinated pathways, and specific prioritization rules. These structural characteristics influence referral patterns, access to advanced support therapies, and transplant allocation. Comparative studies from countries with different PH networks and transplant systems would therefore be valuable to assess the generalizability of our findings.

By combining data from two national registries, this study provides a unique long-term perspective on LT for PH in France. Over the past 16 years, advances in medical therapy, risk assessment, and perioperative management have significantly shaped patient selection and outcomes. Despite the overall increase in lung transplants, Group 1 PAH maintained a stable representation, suggesting preserved and equitable access. The decline in HLT, the increasing age of recipients, and the reduction in high-priority transplantation, while maintaining one-year survival, reflect evolving practices driven by more accurate risk prediction and increasingly individualized decision-making. Yet, an important gap persists between guideline recommendations and real-world listing behaviors, highlighting the need to integrate clinical dimensions that extend beyond formal risk scores, particularly those influencing individual access to transplantation. Altogether, these results underscore the complexity of optimally timing transplantation in PAH and the need for ongoing innovation in treatment strategies, risk assessment, and patient evaluation pathways.

## CRediT authorship contribution statement

Conceptualization: MC. Luong, P. Pradère, J. Le Pavec, D. Horeau-Langlard and L. Savale. Data analysis: P. Pradère. Figure and table development: P. Pradère. Writing – original draft: MC. Luong and P. Pradère. Writing – review and editing: MC. Luong P. Pradère, L. Savale. Collecting data: MC. Luong and P. Pradère. Review: J Issard, B. Coiffard, S. Renard, D. Boulate, B. Renaud-Picard, A. Olland, T. Villeneuve, X. Demant, JM. Maury, D. Mitilian, G. Dauriat, A. Boucly, O. Sitbon, X. Jaïs, D. Montani, M. Humbert, E. Fadel, O. Mercier.

## Declaration of Competing Interest

The authors declare that they have no known competing financial interests or personal relationships that could have appeared to influence the work reported in this paper.

## Footnotes

Appendix A Supplemental data associated with this article can be found in the online version at doi:10.1016/j.jhlto.2026.100675.
