# Supplementary material for: Evolving profile and outcomes of patients with pulmonary arterial hypertension undergoing lung transplantation
Source: JHLT Open. 2026 Sep 3;14:100675. doi: 10.1016/j.jhlto.2026.100675 (PMC13597181; doi:10.1016/j.jhlto.2026.100675)
Supplement: Supplementary file 1 [file mmc1.docx]

**Evolving Profile and Outcomes of Patients with Pulmonary Arterial Hypertension Undergoing Lung Transplantation**

Pauline Pradère^1,2^, Marie Charlotte Luong, MD^3^*; Jérôme Le Pavec, MD, PhD^1,2^; Justin Issard, MD^1,4^; Benjamin Coiffard, MD, PhD^5,6^; Sebastien Renard, MD^7^; David Boulate, MD, PhD^8^, ^,^ Benjamin Renaud-Picard, MD, PhD^9,10^; Anne Olland, MD, PhD^11,12^; Thomas Villeneuve, MD^13,14^; Xavier Demant, MD^15^; Jean Michel Maury, MD^16,17^; Eva Chatron, MD^18^; Delphine Mitilian, MD^4^; Gaëlle Dauriat, MD^1^; Athénaïs Boucly, MD, PhD^19^; Olivier Sitbon, MD, PhD^19^; Xavier Jaïs, MD^19,^ David Montani, MD, PhD^19^;Marc Humbert, MD, PhD^19^; Elie Fadel, MD, PhD^1,4^; Olaf Mercier, MD, PhD^1,4^; Delphine Horeau-Langlard, MD^3&^, Laurent Savale, MD, PhD^19^

^1^ Department of Respiratory Medicine, Marie Lannelongue Hospital, Paris Saint Joseph Hospital, Le Plessis Robinson, France

^2^ INSERM UMR-S1358, Pulmonary Hypertension: Pathophysiology and Novel Therapies, Marie Lannelongue Hospital, Le Plessis Robinson, France

^3^ Department of Respiratory Medicine, CHU Laennec, Nantes, France

^4^ Thoracic Surgery Department, Marie Lannelongue Hospital, Paris Saint Joseph Hospital, Le Plessis Robinson, France

^5^ Department of Respiratory Medicine and Lung Transplantation, Aix Marseille University, APHM, Hôpital Nord, Marseille, France.

^6^ Aix Marseille University, INSERM, INRAE, C2VN, Marseille, France

^7^ Department of Cardiology, Hôpital La Timone, Marseille, France

^8^ Department of Thoracic and Oesophageal Surgery and Lung Transplantation, Aix-Marseille University, APHM, Hôpital-NordMarseille, France.

^9^ Strasbourg Lung Transplant Program, Strasbourg University Hospital, Strasbourg, France.

^10^ INSERM UMR 1260, University of Strasbourg, Strasbourg, France

^11^Thoracic Surgery and Lung Transplantation Department, University Hospital Strasbourg, Strasbourg, France.

^12^ Research Unit 1260, Regenerative Nanomedicine INSERM UMR 1260, Université de Strasbourg CRBS, Strasbourg, France.

^13^ Department of Respiratory Medicine, University Hospital, Toulouse, France

^14^ INSERM UMR 1291, Toulouse Institute for Infectious and Inflammatory Diseases (Infinity), Université de Toulouse, France

^15^ Department of Respiratory Medicine, Haut-Lévêque Hospital, Bordeaux, France

^16^ Department of Thoracic Surgery, Lung and Heart-Lung Transplantation, Hôpital Louis Pradel, Hospices Civils de Lyon, Lyon, France.

^17^ EA 3738 CICLY, Université Claude Bernard Lyon 1, Lyon, France. ^14^ Physiopathology and Epidemiology of Respiratory Diseases, UMR1152 INSERM and Universite de Paris, Paris, France

^18^ Hospices Civils de Lyon, Hôpital Louis Pradel, Department of Respiratory Medicine, Lyon, France.

^19^ Université Paris–Saclay, Inserm UMR_S 1358 (HPPIT), Department of Respiratory and Intensive Care Medicine (FHU André Cournand, ERN-LUNG), Hôpital Bicêtre (AP-HP), Le Kremlin Bicêtre, France

**ONLINE DATA SUPPLEMENT**

**Supplemental Figure**

**Supplemental Figure 1. Proportion of patients managed with pre- and post-operative ECMO**

Abbreviation: ECMO for extracorporeal membrane oxygenation.

**Supplemental Figure 2. Proportion of double lung transplantation and heart-lung transplantation**

**Supplemental Tables**

**Supplemental Table 1. One and five-year post-transplant survival according to pre-transplant disease**

|  | **Period 1**  **2008-2015** | **Period 2**  **2016-2023** |
| --- | --- | --- |
| **Group I PAH***  One year, median [Q1-Q3]  Five years, median [Q1-Q3] | 75% [67–84%]  60% [50-70%] | 76% [69–83%]  63% [55-72%] |
| **Cystic fibrosis^1^**  One year, median [Q1-Q3]  Five years, median [Q1-Q3] | 86% [83-88%]  71% [67-74%] | 88% [84- 90%]  76% [72-80%] |
| **COPD-emphysema**  One year, median [Q1-Q3]  Five years, median [Q1-Q3] | 80% [77-83%]  61% [57-64%] | 86% [84-88%]  66% [62-70%] |
| **Lung fibrosis**  One year, median [Q1-Q3]  Five years, median [Q1-Q3] | 66% [62-70%]  44% [39-48%] | 80% [77-82%]  54% [49-59%] |
| *p value* | *p<0.001* | *p<0.001* |

*Congenital heart disease excluded ^1^And other bronchiectasis

Abbreviations: PAH for Pulmonary Arterial Hypertension and COPD for Chronic Obstructive Pulmonary Disease.

**Supplemental Table 2. Comparison of patient outcomes according to the transplant center**

|  | **National Reference center***  **n=183** | **Other**  **centers^#^**  **n= 58** | **Missing data**  **n (%)** | **P value** |
| --- | --- | --- | --- | --- |
| **Age at transplant, years,** median [Q1-Q3] | 43.7 [30.8-53.1] | 49.2 [37.8-57.5] | 0 (0) | <0.01 |
| **Age at transplant ≥ 50 years,** n (%) | 64 (35) | 27 (47) | 0 (0) | 0.11 |
| **Sex, female,** n (%) | 119 (65) | 36 (62) | 0 (0) | 0.76 |
| **Type of PAH,** n (%)   - Idiopathic - Heritable - PVOD - Drug-induced PAH - Connective tissue disease - PoPH associated with HIV | 51 (28)  46 (25)  47 (26)  10 (6)  23 (13)  1 (1) | 20 (35)  11 (19)  11 (19)  1 (2)  15 (26)  0 (0) | 0 (0) | 0.14 |
| **Haemodynamic**   - mPAP, mm Hg, median [Q1-Q3] - PAWP, mm Hg, median [Q1-Q3] - RAP, mm Hg, median [Q1-Q3] - CI, l/min/m, median [Q1-Q3] - PVR, WU, median [Q1-Q3] | 55.0 [47.0-65.0]  8.0 [6.0-11.0]  7.0 [5.0-12.0]  2.8 [2.2-3.3]  10.0 [7.0-13.8] | 46.0 [37.0-54.0]  8.0 [6.0-11.8]  7.0 [3.3-9.8]  2.6 [2.1-3.3]  8.5 [5.0-11.6] | 4 (2)  9 (4)  9 (4)  8 (3)  9 (4) | <0.01  0.68  0.19  0.51  0.04 |
| **High priority list,** n (%) | 94 (51) | 20 (36) | 0 (0) | 0.05 |
| **Evaluated and bridged to lung transplant in ICU^1^,** n (%) | 32 (18) | 4 (7) | 0 (0) | 0.09 |
| **ECMO as bridge to transplant,** n (%) | 22 (12) | 4 (7) | 8 (6) | 0.74 |
| **Type of transplant, n (%)**   - Heart-lung transplantation - Double-lung transplantation | 22 (12)  161 (88) | 6 (11)  50 (89) | 0 (0) | 0.83 |
| **One year post-transplantation survival** | 74% (95CI: 68%-81%) | 79% (95CI: 68%-91%) | 0 (0) | 0.8 |

*The National Reference center for transplantation for PAH refers to Marie Lannelongue Hospital (Le Plessis Robinson).

#The other centers are Hôpital Haut-Levêque at Bordeaux (n=15), Hôpital Louis-Pradel at Lyon (n=14), Hôpital Laennec at Nantes (n=12), Nord Hospital at Marseille (n=9), Hôpital Civil at Strasbourg (n=6), Hôpital Rangueil at Toulouse (n=2).

Abbreviations are HIV for human immunodeficiency virus, PAH for pulmonary arterial hypertension, PVOD, for pulmonary veno-occlusive disease, PoPH for porto-pulmonary pulmonary hypertension, mPAP for mean pulmonary arterial pressure, PAWP for pulmonary arterial wedge pressure, RAP for right atrial pressure, CI for cardiac index, PVR for pulmonary vascular resistance, WU for Wood Units, ECMO for extracorporeal membrane oxygenation.
